# Genomic characterization of NDM-1 and 5, and OXA-181 carbapenemases in uropathogenic *Escherichia coli* isolates from Riyadh, Saudi Arabia

**DOI:** 10.1371/journal.pone.0201613

**Published:** 2018-08-15

**Authors:** Moataz Abd El Ghany, Hazem Sharaf, Mohamed H. Al-agamy, Atef Shibl, Grant A. Hill-Cawthorne, Pei-Ying Hong

**Affiliations:** 1 Westmead Institute for Medical Research, The University of Sydney, Sydney, Australia; 2 Marie Bashir Institute for Infectious Diseases and Biosecurity, The University of Sydney, Sydney, Australia; 3 Interdisciplinary PhD Program in Genetics, Bioinformatics, and Computational Biology, Virginia Polytechnic Institute and State University, Blacksburg, VA, United States of America; 4 Department of Pharmaceutics and Microbiology, College of Pharmacy, King Saud University, Riyadh, Saudi Arabia; 5 Department of Microbiology and Immunology, Faculty of Pharmacy, Al-Azhar University, Cairo, Egypt; 6 Microbiology and Immunology Department, College of Medicine, Alfaisal University, Riyadh, Saudi Arabia; 7 School of Public Health, The University of Sydney, Sydney, Australia; 8 Water Desalination and Reuse Center, Environmental Science and Engineering, Division of Biological and Environmental Science and Engineering, King Abdullah University of Science and Technology (KAUST), Jeddah, Saudi Arabia; Zhejiang University, CHINA

## Abstract

Urinary tract infections (UTIs) associated with *Escherichia coli* are a growing threat with an increase in the prevalence of multidrug resistant (MDR) strains, particularly ß-lactamase producers, occurring globally. We investigated the presence of carbapenem-resistant uropathogenic *E*. *coli* clones in community-acquired UTIs in Riyadh, Kingdom of Saudi Arabia (KSA) to identify the virulence and resistance structures of the resistant clones and relate the isolates to those circulating globally. A combination of comparative genomics and phenotypic approaches were used to characterize ten MDR-uropathogenic *Escherichia coli* isolates recovered from UTI patients in Riyadh between November 2014 and January 2015. We report the presence of NDM-1 and 5, and OXA-181 in carbapenem-resistant UPEC strains from Riyadh, KSA. Single nucleotide polymorphism analyses demonstrated that these ten isolates fell into four phylogenetically distinct clades within the UPEC phylogeny. Comparative genomic analyses indicate that these diverse clones could be distinguished according to their multilocus sequencing type (MLST), serology, and virulence and antimicrobial gene architectures. These clones include the *bla*_NDM-1_ carrying isolates of the globally predominant MDR ST131 and ST69 types, previously identified as one of the most common UPEC strains in KSA. This is in addition to clones of ST23Cplx (ST410) and ST448Cplx (ST448) that have likely evolved from common intestinal strains, carrying copies of ß-lactamase genes including *bla*_NDM-5,_ *bla*_CTX-M-15_, *bla*_TEM-1,_ *bla*_CMY-42,_ *bla*_OXA-1_ and *bla*_OXA-181._ These data have identified an emerging public health concern and highlight the need to use comprehensive approaches to detect the structure of MDR *E*. *coli* populations associated with community-acquired UTIs in KSA.

## Introduction

Uropathogenic *Escherichia coli* (UPEC) is a subset of extra-intestinal pathogenic *E*. *coli* (ExPEC) that are capable of colonizing the urogenital tract and are responsible for 70–90% of community-acquired urinary tract infections (UTIs) and ~50% of nosocomial UTIs [1]. It has been estimated that ~20% of women over the age of 18 years suffer from at least one episode of UTI in their lifetime [2]. UPEC cause UTIs through the colonization of the human gastrointestinal tract, followed by infection of the urogenital tract, and subsequent disease establishment promoted by bacteria-specific traits (virulence factors) and host-related factors [3]. Recently, UTIs have been associated with ExPEC strains originating from diverse sources that include foodborne, animal sources and even through sexual transmission [4,5].

UPEC are a heterogeneous group of strains that are commonly associated with particular O-serogroups and specific virulence profiles. UPEC virulence factors have likely been acquired through horizontal transfer and are mainly encoded on pathogenicity islands [6]. UPEC invade and colonize the urinary tract through the expression of fimbrial adhesins including type 1 (*fim*), P (*pap*), S (*sfa*), and F1C (*foc*) fimbriae [7], and use flagella to move via the ureters to the kidney (leading to pyelonephritis) and to enter the bloodstream (leading to urosepticemia) [8]. UPEC possess multiple iron-acquisition systems that are used to transfer the iron required for bacterial cellular processes across the bacterial membrane [9]. UPEC produce several toxins including α-hemolysin (HlyA), cytotoxic necrotizing factor 1 (CNF1) and type V autotransporters that promote bacterial dissemination through the disruption of cellular integrity [10]. UPEC have the capacity to either disrupt host inflammatory signaling or mask immunogenic markers, allowing them to evade the host immune response [10].

The increased prevalence of antimicrobial-resistant (AMR) UPEC isolates has been identified globally. For instance, an increasing incidence of UPEC strains resistant to trimethoprim-sulfamethoxazole and fluoroquinolones has been reported [11,12]. This is in addition to the emergence of UPEC strains resistant to plasmid-mediated AmpC β-lactamases (e.g. CMY), extended spectrum β-lactamases (ESBLs e.g. CTX-M), and carbapenemases (e.g. NDM) [13]. The emergence of single- and multi-drug resistant isolates makes treating these infections increasingly challenging [14].

The highly virulent and common MDR sequence type 131 (ST131) UPEC strain now causes the majority of extraintestinal infections in humans and has disseminated globally [15]. This strain contains mobile elements that confer resistance to most of the critically important antimicrobial classes, including fluoroquinolones and third- and fourth-generation cephalosporins. This is alarming when one considers the high probability of horizontal gene transfer occurring among *E*. *coli* serovars.

Here we used a combination of molecular, whole genome sequencing and phenotypic approaches to characterize ten clinical isolates of MDR UPEC from two hospitals in Riyadh, KSA collected between November 2014 and January 2015. The analyses identified four UPEC clones with different evolutionary origins that are characterized by sequence type complex (ST Cplx) and distinct antimicrobial resistance and virulence architectures.

## Materials and methods

### Ethical statements

All UPEC isolates described in this study were originally recovered for the purpose of diagnosis and were not experimental in nature. All clinical data were anonymized and unlinked and therefore informed consent was not necessary.

### Selection of isolates

Ten serial UPEC isolates that displayed an imipenem minimum inhibitory concentration (MIC) ≥ 1μg/ml were recovered from inpatients with bacteremias from the intensive care units (ICUs) at two hospitals in Riyadh, KSA during the monitoring period of November 2014 to January 2015. All ten patients had been suffered from symptoms of UTI and were admitted directly to ICU from the emergency department and therefore these UPEC infections are thought to be community-acquired.

### Antimicrobial resistance profiling of UPEC isolates

MICs of piperacillin (PIP), piperacillin/tazobactam (TZP), cefotaxime (CTX), ceftazidime (CAZ), ceftazidime/ceftazidime+clavulanic acid (CAZ/CAL), cefoperazone (CP), cefoperazone/sulbactam (CPS), cefepime (FEP), cefoxitin (FOX), cefotetan (CTT), cefotetan/cefoxitin (CTT/CXT), aztreonam (ATM), imipenem/ imipenem+EDTA (IMI/IMD), imipenem (IMI), meropenem (MRP), ertapenem (ERT), doripenem (DOR), temocillin (TM), ciprofloxacin (CI), gentamicin (GM), and amikacin (AK), were determined by Etest strips (bioMérieux, Marcy l’Etoile, France). The MICs of colistin (COL) and tigecycline (TGC) were determined by microbroth dilution method, while the MIC of fosfomycin (FOS) was determined by agar dilution method using Mueller-Hinton agar supplemented with 25 μg/ml of glucose-6-phosphate. Interpretation was based on Clinical and Laboratory Standards Institute (CLSI) criteria [CLSI, 2014] for all antibiotics except for TM, COL, TGC and FOS. British Society on Antimicrobial Chemotherapy (BSAC) breakpoints were used for these four antimicrobials [BSAC, 2015] [16]. *E*. *coli* ATCC 25922 strain was used as the control. In addition, UPEC isolates were characterized molecularly to detect the carbapenemase markers using qualitative multiplex PCR as previously described [17].

### Preparation of genomic and plasmid DNA

For each isolate, chromosomal and plasmid DNA were extracted using Promega Wizard genomic DNA purification kit (Promega, Madison, WI, USA) and QIAfilter Plasmid Midi Kit (Qiagen, Hilden, Germany), respectively. The extraction of DNA was performed according to the manufacturers’ instructions. No plasmid DNA was extracted for isolate UPEC-RIY-9.

### Preparation of Illumina sequencing libraries

For each isolate, chromosomal and plasmid (except UPEC-RIY-9) DNA were used to prepare paired-end Illumina libraries. Chromosomal libraries with a 300 bp insert size were prepared using Nextera DNA Library Prep Kit (Illumina Inc.) and sequenced on an Illumina HiSeq2000 platform. Plasmid libraries with a 300 bp insert size were prepared using Nextera DNA Library Prep Kit (Illumina Inc.) and sequenced on an Illumina MiSeq platform using the MiSeq 500-cycle kit V3 (Ilumina Inc.). Both chromosomal and plasmid sequencing libraries were sequenced at the KAUST Bioscience Core Laboratory.

### Phylogenomic analysis

The assembled reads of the chromosomes from the isolates sequenced in this study, UPEC reference genomes and publicly available draft genomes from Petty et al. [15] and Salipante et al. [18] (accession numbers are given in S1 Table) were then aligned to generate a core genome alignment using Parsnp v1.2 from the Harvest suite [19]. Whole genome SNPs derived from the core alignment were concatenated and processed in FastTree2 [20] to generate a maximum-likelihood phylogenetic tree, with branch support values calculated using the Shimodaira-Hasegawa (SH) test [21].

### Genome assembly, annotation and comparative genomics

The draft genomes and plasmid assemblies were generated using a local pipeline. Briefly, paired-end Illumina reads were trimmed using Trimmomatic [22] to exclude low quality reads and then were binned to plasmids and chromosomes using BBMap (https://sourceforge.net/projects/bbmap/). The trimmed paired-end Illumina reads (> Q30) were assembled *de novo* using the SPAdes assembler pipeline [23]. SPAdes assembler was run with a coverage cutoff of 5.0 and scaffolds shorter than 400bp were filtered out. The assemblies were then corrected and broken down using REAPR [24]. The broken assemblies underwent another round of scaffolding with SSPACE [25] and the gaps were filled using GapFiller [26]. Additionally, the assembly was finished using IMAGE and corrected using four iterations of ICORN2 [27]. Scaffolds were reordered against *E*. *coli* SE15 (Genbank accession NC_013654) and annotation was performed using the Prokka pipeline [28]. Core and pan genome analyses were conducted on the predicted proteins using a protein identity threshold of 95% and 90% core clustering limit with Roary [29]. Comparative analysis was performed for the UPEC isolates from Riyadh and the seven publicly available UPEC reference genomes. Region of differences (RODs) were defined as insertions, deletions or rearrangements in any of the UPEC genomes from Riyadh in comparison to the bacteremic UTI- associated genome of *E*. *coli* CFT073 (accession number AE014075). These regions were identified by using a whole genome alignment using progressiveMauve [30]. The RODs were then imported into the comparative genome analysis that was visualized in BRIG [31]. An E value of 1x10^-5^ was used in the BLASTn comparison against the reference *E*. *coli* CFT073 for the BRIG visualization.

### *In silico* characterization of UPEC isolates

Virulence factors were identified in the chromosomes and plasmid assemblies using a local BLAST against the January 2016 release of the Virulence Factors of Bacterial Pathogens database (VFDB) [32] and VirulenceFinder 1.5 [33]. The multi-locus sequence types (MLSTs), AMR genotypes and the serotypes of the studied isolates were extracted from the genomic data using the MLST 1.8 database, [34] ResFinder 2.1 [35] and SerotypeFinder 1.1, [36] respectively. ST complexes were assigned according to *E*. *coli* MLST scheme (http://enterobase.warwick.ac.uk/species/ecoli/search_strains) [37]. The plasmid multi-locus sequence typing and AMR genotypes were extracted from the plasmid assemblies using the pMLST 1.4 web tool [38] and ResFinder 2.1, [35] respectively.

### Accession numbers

The chromosomal and plasmid raw sequencing data generated in this study have been submitted to the European Nucleotide Archive (ENA). Study accession number PRJEB17503. The accession numbers for the chromosomal sequences are ERR1720656 (UPEC-RIY-1), ERR1720657 (UPEC-RIY-2), ERR1720658 (UPEC-RIY-3), ERR1720659 (UPEC-RIY-4), ERR1720660 (UPEC-RIY-5), ERR1720661 (UPEC-RIY-6), ERR1720662 (UPEC-RIY-7), ERR1720663 (UPEC-RIY-8), ERR1720664 (UPEC-RIY-9) and ERR1720665 (UPEC-RIY-10). The accession numbers for the plasmid sequences are ERS1443905 (UPEC-P-RIY-1), ERS1443906 (UPEC-P-RIY-2), ERS1443907 (UPEC-P-RIY-3), ERS1443908 (UPEC-P-RIY-4), ERS1443909 (UPEC-P-RIY-5), ERS1443910 (UPEC-P-RIY-6), ERS1443911 (UPEC-P-RIY-7), ERS1443912 (UPEC-P-RIY-8), and ERS1443913 (UPEC-P-RIY-10).

## Results

### Molecular and phenotypic characterization of carbapenem-resistant UPEC isolates from Riyadh, KSA

Phenotypically, all of the studied isolates exhibited increased resistance to major types of antimicrobial agents including ß-lactams (third- and fourth-generation cephalosporins), fluoroquinolones and aminoglycosides (Table 1). In addition, 100%, 60%, 50% and 40% of the studied UPEC isolates were found to be resistant to imipenem, ertapenem, meropenem and doripenem, respectively. 20% of the isolates were intermediately resistant to meropenem and doripenem. All isolates were found to be sensitive to colistin, fosfomycin and tigecycline. The isolates were screened molecularly using multiplex PCR to characterize the ESBL *bla*_CTX-M-15_ gene and the carbapenemase resistance genes; *bla*_NDM_, *bla*_OXA-181_, *bla*_KPC_ and *bla*_VIM_. *bla*_CTX-M-15_ was detected in all of the UPEC isolates. *bla*_NDM_ was detected in all of the isolates except UPEC-RIY-3 to UPEC-RIY-6 where *bla*_OXA-181_ was detected instead.

**Table 1:** Antimicrobial resistance phenotypes of UPEC isolates.

ID	MIC (mg/L)																								Carbapenemase genes*	pMLST Profile	
	PIP	TZP	CTX	CAZ	CAZ/CAL	CP	CPS	FEP	FOX	CTT	CTT/CXT	ATM	IMI/IMD	IMI	MRP	ERT	DOR	TM	CI	GM	AK	COL	TGC	FOS		IncF	Incl1
**UPEC-RIY-1**	>256	>256	>256	>256	>32/>4	>256	>256	>256	>256	>32	>32	>256	64/<1	64	2	6	4	12	>32	0.5	12	<0.016	0.25	1.5	NDM-1	F1:A1:B20	
**UPEC-RIY-2**	>256	>256	>256	>256	>32/>4	>256	>256	>256	>256	>32	>32	>256	64/<1	64	4	6	2	2	>32	1.5	32	1	0.38	2	NDM-1	F1:A2:B20	
**UPEC-RIY-3**	>256	>256	>256	>256	>32/>4	>256	>256	>256	>256	>32	>32	>256	64/<1	64/<1	0.75	0.75	0.25	12	>32	24	4	1	0.125	2	OXA-181	F48:A1:B49	Unknown#
**UPEC-RIY-4**	>256	>256	>256	>256	>32/>4	>256	>256	>256	>256	>32	>32	>256	64/<1	64/<1	2	0.75	1	8	>32	24	4	1	0.125	1.5	OXA-181	F48:A1:B49	Unknown#
**UPEC-RIY-5**	>256	>256	>256	>256	>32/>4	>256	>256	>256	>256	>32	>32	>256	64/<1	64/<1	0.75	0.5	1	4	>32	8	3	1	0.25	1.5	OXA-181	F48:A1:B49	Unknown#
**UPEC-RIY-6**	>256	>256	>256	>256	>32/>4	>256	64	>256	>256	>32	>32	>256	64/<1	64/<1	0.25	0.75	0.38	8	>32	192	6	1	0.25	1.5	OXA-181	F48:A1:B49	Unknown#
**UPEC-RIY-7**	>256	>256	>256	>256	>32/>4	>256	>256	>256	>256	>32	>32	48	12/<1	12	6	12	8	12	>32	256	>256	1	0.25	1.5	NDM-5	F31:A4:B1	Unknown§
**UPEC-RIY-8**	>256	>256	>256	>256	>32/>4	>256	>256	>256	>256	>32	>32	64	128/<1	128	>32	32	>32	32	>32	192	>256	0.75	0.25	0.75	NDM-5	F31:A4:B1	Unknown§
**UPEC-RIY-9**	>256	>256	>256	>256	>32/>4	>256	>256	>256	>256	>32	>32	16	48/<1	48	4	8	2	16	>32	256	>256	1	0.38	1.5	NDM-5	ND	ND
**UPEC-RIY-10**	>256	>256	>256	>256	>32/>4	>256	>256	>256	>256	>32	>32	>256	>256/<1	96	24	24	8	48	>32	512	>256	1	0.38	1.5	NDM-5	F31:A4:B1	Unknown§

PIP piperacillin, TZP tazobactam, CTX cefotaxime, CAZ ceftazidime, CAZ/CAL ceftazidime/ clavulanic acid, CP cefoperazone, CPS cefoperazone/sulbactam, FEP cefepime, FOX cefoxitin, CTT cefotetan, CTT/CXT cefotetan/cefoxitin, ATM aztreonam, IMI/IMD imipenem/imipenem+EDTA, MRP meropenem, ERT ertapenem, DOR doripenem, TM temocillin, CI ciprofloxacin, GM gentamicin, AK amikacin, COL colistin, TGC tigecycline, FOS fosfomycin. Values highlighted in gray: resistant, values highlighted in yellow: intermediate, and non-highlighted values: sensitive.

*Characterization of the resistance genes was performed using PCR and the resistance alleles extracted from the genomic data. The IncI1 profiles are provided;

^#^ (*ardA*_0/ *pilL*_3/ *repI1*_4/ *sogS*_0/ *trbA*_15) and

^§^ (*ardA*_5*/ pilL*_0*/ repI1*_4*/ sogS*_0*/ trbA*_15). ND; not done (no plasmid DNA was extracted from UPEC-RIY-9).

### Characterization of plasmid-mediated AMR genotypes in UPEC isolates

**Fig 1:**
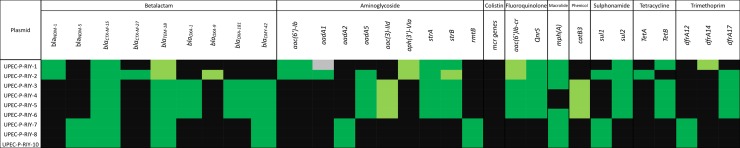
The distribution of plasmid genes associated with antimicrobial resistance among UPEC isolates from KSA. Heat map showing the antimicrobial resistance genotypes identified in the plasmids contained in the UPEC isolates from Riyadh, KSA. The resistance genes were identified by ResFinder 1.5 using BLAST analysis against the acquired resistance sequences of ResFinder 1.5 database. Black: no sequence matching, dark green: perfect match (100% of sequence identity and 100% of sequence length), pale green: match (<100% of sequence identity and 100% of sequence length), grey: weak match (90–100% of sequence identity and 80–90%of sequence length). No plasmid sequencing data was generated for UPEC-RIY-9.

The phenotypic AMR profiles of UPEC isolates from KSA were consistent with AMR genotypes predicted *in silico* from chromosomal and plasmid assemblies. The isolates contained plasmids that harbour resistance genes to a plethora of AMR classes including ß-lactamases, aminoglycosides, fluoroquinolones, macrolides, phenicol, sulfonamides, tetracyclines and trimethoprim. The antimicrobial resistant genotypes identified in the plasmids contained in the studied isolates are shown in Fig 1.

The isolate UPEC-RIY-1 (F1:A1:B20) carried genotype 1 of *bla*_NDM_. This is in addition to ceftazidime and TEM-hydrolysing genes *bla*_CTX-M-15_ and *bla*_TEM-1_. A similar resistance genotype was identified in the isolate UPEC-RIY-2 (F1:A2:B20) with two extra carbapenem-hydrolyzing genes *bla*_CTX-M-27_ and *bla*_OXA-9_ present. Interestingly, the isolates UPEC-RIY-3 to UPEC-RIY-6 (F48:A1:B49 and novel IncI1 pMLST), carried copies of *bla*_CTX-M-15_, *bla*_TEM-18,_ *bla*_CMY-42_ and two carbapenem-hydrolysing class D ß-lactamase genes *bla*_OXA-1_ and *bla*_OXA-181_. This is in addition to aminoglycoside (*aadA5*, *aac(3)-IId*, *strA* and *strB*) and trimethoprim (*dfrA17*) resistance genes. The isolates UPEC-RIY-[7–8] and UPEC-RIY-10 carried three carbapenemase-encoding genes: *bla*_NDM-5,_ *bla*_TEM-18_ and *bla*_CMY-42_. This is in addition to resistance genes against aminoglycosides (*aadA2* and *rmtB*), sulfonamides (*sul1*) and trimethoprim (*dfrA12*).

All of the sequenced UPEC strains from Riyadh, except isolates UPEC-RIY-[7–8] and UPEC-RIY-10 (F31:A4:B1 and novel IncI1 pMLST), carried the fluoroquinolone-resistance genes *aac(6')Ib-cr* and *QnrS1* and tetracycline resistance gene *tetB* (*tetA* was identified in UPEC-RIY-2). Similarly, the sulfonamide-resistant gene *sul2* was identified in all isolates except UPEC-RIY-[7–8] and UPEC-RIY-10.

**Fig 2:**
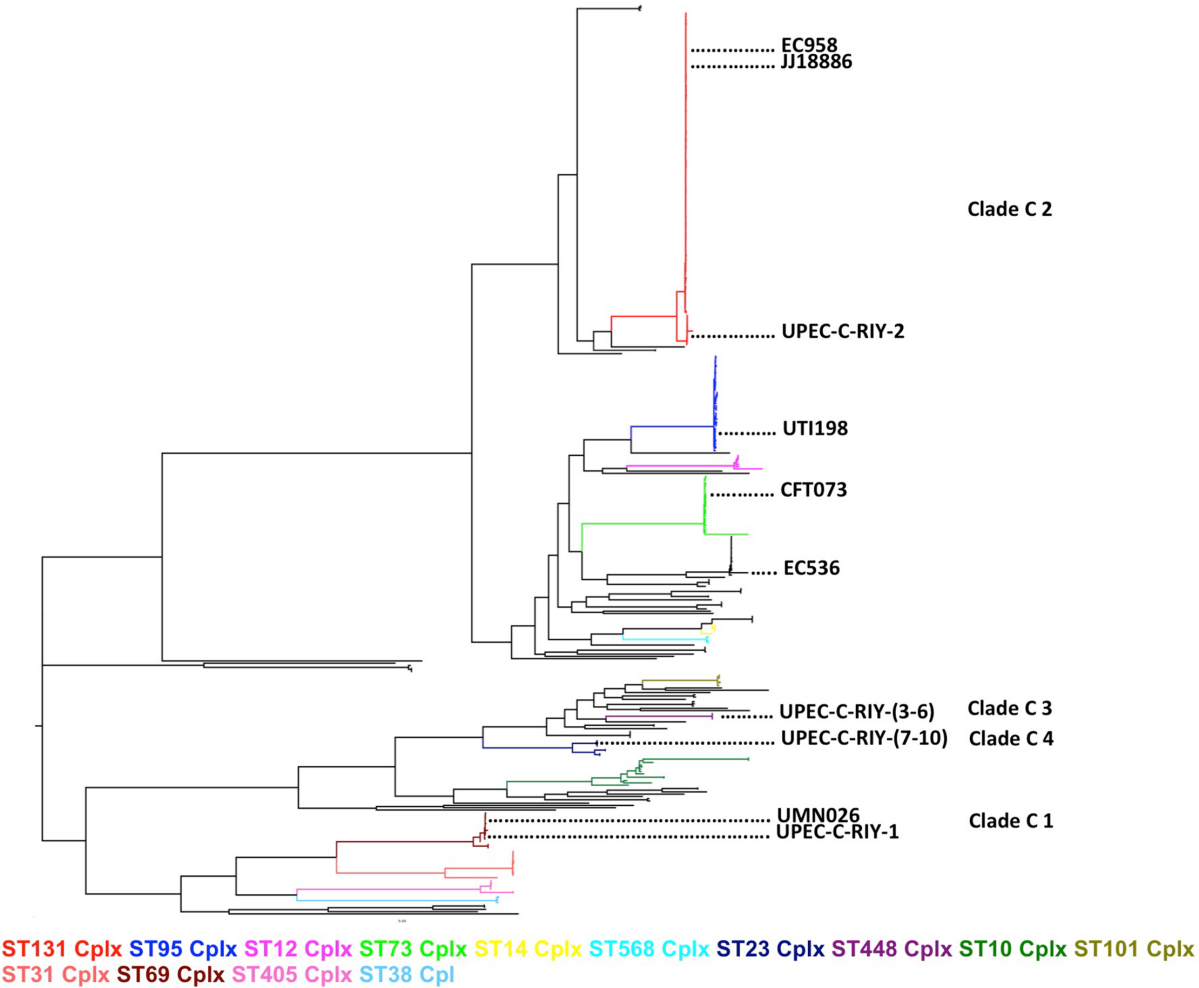
Phylogenomic distribution of publicly available UPEC isolates with KSA isolates from this study. A mid-point rooted SNP-based maximum likelihood phylogenomic tree of the MDR UPEC isolates from KSA, UPEC reference genomes and publicly available *E coli* genomes associated with UTI from Petty et al. [15] and Salipante et al. [18] (accession numbers are given in S1 Table). The major phylogenomic clades were colored according to MLST Cplx with the positions of UPEC reference genomes indicated.

The plasmids contained in the UPEC isolates form Riyadh, KSA could be categorized into four major pMLST types that correlated with AMR signatures and phylogeny (Table 1 and Fig 2).

### Characterization of chromosomal-mediated quinolone resistance in UPEC isolates

It has been well established that point mutations in DNA gyrase and topoisomerase genes are associated with quinolone resistance in Gram-negative bacteria [39–41]. The presence of point mutations in the chromosomal genes encoding fluoroquinolone targets that include DNA gyrase (*gyrA* and *gyrB*) and topoisomerase (*parC* and *parE*) were checked in the studied UPEC isolates using ResFinder 1.5. All of the UPEC isolates from KSA were characterised by point mutations in chromosomal genes *gyrA*, *parC* and *parE* but not *gyrB* (S2 Table). The SNP patterns identified in these chromosomal genes (S2 Table) distinguish the isolates into four categories that correlated with pMLST and phylogeny. UPEC-RIY-[7–10], lacking plasmid-quinolone resistant genes, shared point mutations in chromosomal genes *gyrA* (S83L and D87N) and *parC* (S80I) and therefore were phenotypically resistant to fluoroquinolones (S2 Table and Table 1). The distribution of AMR plasmid and chromosomal genes identified in UPEC isolates from KSA are listed (S3 Table).

### Phylogenomic analysis of UTI-associated *E*. *coli* isolates from KSA

The UPEC genomes generated in this study and 393 published genomes [15,18], representing a moderately global collection of UPEC, were aligned using parSNP [19] against 965,448 bp of sequence present in all of the genomes. A total of 71,521 SNPs found against the core alignment were extracted and concatenated to construct a maximum likelihood phylogeny. The available UPEC sequences clustered into multiple clades that were consistent with their sequence type complexes (STCplx) (Fig 2). The majority of UPEC isolates belonged to five major clades that included ST complexes ST131, ST95, ST69, ST73 and ST10 (Fig 2). Previous large-scale genomic epidemiology studies have shown that UPEC strains of ST complexes ST131, ST95, ST69 and ST73 are the predominant strains associated with common and bacteremic UTIs [15,18,42,43].

The UPEC isolates from Riyadh, KSA clustered into four distinct clades designated C1-4, of which C1 and C2 were among the major UPEC clades [15,18,42,43], while C3 and C4 clades only comprised isolates from KSA. The *bla*_NDM-1_ resistant isolate UPEC-RIY-1 of serotype O17/O77:H18 and other ST69 Cplx strains; including the reference strain UMN026 (ST597), clustered in clade C1. The other *bla*_NDM-1_ resistant isolate UPEC-RIY-2 of serotype O16:H5 and the globally predominant multidrug resistant ST131Cplx strains clustered in clade C2. Although, UPEC strains of ST69 (clade C1) and ST131 Cplx (clade C2) have been identified among the most common MDR UPEC strains in KSA, [44] this is the first report of *bla*_NDM-1_ resistant UPEC isolates in the country.

The ST448 Cplx strains, sharing a novel O-antigen and flagellar antigen H19, clustered in clade C3 while the the *bla*_NDM-5_ resistant isolates of ST23Cplx (ST410) sharing the serotype O8:H21 clustered in clade C4. Interestingly, the clade C3 isolates (UPEC-C-RIY3–6) had a close evolutionary relationship with the JSGI01 isolate (4491 SNPs different) of ST453 that has previously been recovered from urine. The clade C4 isolates (UPEC-C-RIY7-10) shared a close evolutionary relationship with ST88 isolates recovered from bacteremias (JSSG01 [1412 SNPs different] and JSSI01 [1412 SNPs different] that share novel O- and H12 antigens) and urinary tracts (JSNE01 [1273 SNPs different] and JSND01 [1274 SNPs different] of serotype O9:H17).

### Comparative analysis of UPEC isolates and virulence architecture

**Fig 3:**
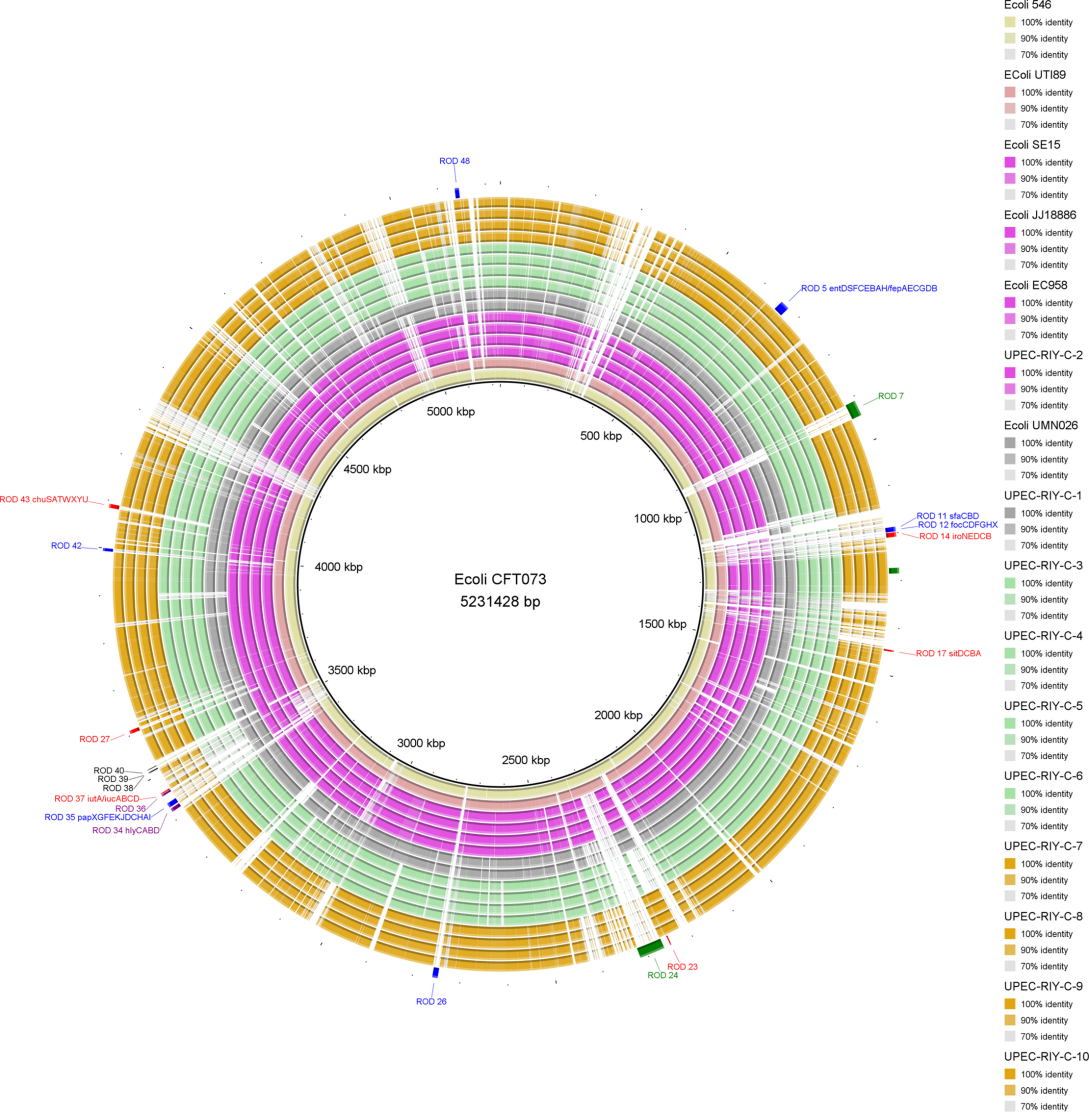
Comparative genomic analysis of UPEC isolates from Riyadh, KSA. The ORFs of the UPEC genomes sequenced in this study and the available reference genomes representing distinct UPEC phylogenetic lineages were compared against the reference genome of *E*. *coli* CFT073 (accession number AE014075). The genomes are displayed in the order of the legend on the right (going from innermost to outermost). The structural variations identified, particularly those associated with key UPEC virulence factors, are displayed on the outermost ring and were coded according to the associated function/origin (red, iron acquisition; blue, colonization factors [fimbriae, pilli]; black, capsule synthesis; purple, toxin and green, phage-related).

Comparative genomic analysis for UPEC isolates from Riyadh and the seven publicly available reference genomes representing distinct evolutionary clades was performed to identify structural variations in the chromosomes of the studied isolates (Fig 3 and S4 Table). Artemis Comparison views showing structural variations identified in the chromosome and virulence architectures (e.g. colonization factors, flagella, toxins and iron acquisition systems) of the UPEC isolates from Riyadh are displayed in S1 Fig. The distribution of virulence genes identified among the UPEC isolates from KSA is detailed in S5 Table.

The studied isolates from Riyadh share key virulence factors required for UPEC pathogenesis, including the type 1 fimbrial pili operon *fimABCDFEGHI* that promotes cellular colonization [45]. The type 1 fimbriae also induce the formation of biofilm-like intracellular bacterial communities (IBCs) that protect bacteria from the host immune response and therapeutic stress [46]. This is in addition to the enterobactin siderophore (*entABCDEFS*) that assists bacterial survival in iron-limited environments such as the bladder [9,47].

Although a high level of diversity with regard to the presence/absence of virulence genes was identified, the virulence architecture of the UPEC isolates from Riyadh correlated with their evolutionary position in the phylogeny (Fig 2 and S5 Table). The *bla*_NDM-1_ resistant isolates UPEC-C-RIY-1 and UPEC-C-RIY-2 belonging to the major UPEC clades of ST69 Cplx (Clade C1) and ST131 Cplx (clade C2) respectively, shared all classical UPEC virulence factors and hence seem to be highly virulent. These isolates share four types of colonization factors (Fim, Pap, SfA, foc) including *papG* that promotes the establishment of infection in the human kidney through the binding of kidney globoside-containing glycolipids [48]. This is in addition to four distinct iron acquisition systems, including enterobactin (*entABCDEFS*), aerobactin (*iutA*), yersiniabactin (*fyuA*) and the heme uptake system (*chuSTUVWXY*), and these were the only isolates that shared a copy of the secreted autotransporter toxin sat (Fig 3). The variations identified in the structure of the iron acquisition systems distinguished the clade C3 and C4 isolates with the former sharing identical deletions in *sitDCBA* (ROD 17), yersiniabactin (ROD 23) and aerobactin (ROD 37). The clade C3 (UPEC-C-RIY-[3–6]) isolates still contain abundant plasmid-borne MDR genes including *bla*_CTX-M-15_, *bla*_TEM-1,_ *bla*_CMY-42_, *bla*_OXA-1_ and *bla*_OXA-181_. This MDR pattern, particularly variants of *bla*_*OXA*,_ has previously been reported in the region and is mainly associated with enterobacterial infections [49].

The analyses provide some evidence that the clade C3 and C4 isolates have evolved from a common ancestor of intestinal origin. All UPEC isolates from Riyadh, except the ST131 Cplx UPEC-C-RIY-2, shared an insertion of ~27 kb at tRNA-Gly (ROD 32) that has previously only been identified in intestinal *E*. *coli* strains (e.g O26:H11 str. 113688, AZ155 and FHIXX). This sequence includes genes encoding for type-III-secretion system proteins associated with cell invasion (*prgK* and *inv*) and flagellar formation (*flhB* and *lfiR*). Both clade C3 (UPEC-C-RIY-[3–6]) and C4 isolates (UPEC-C-RIY-[7–10]) share an insertion of ~8.2 kb at *aapA* (ROD 13) that includes ORFs encoding for O antigen-associated protein. Similarly, these isolates share identical deletions in *kpsE* (ROD 39), *kpsF* (ROD 38) and *kpsS* (ROD 40) that have been associated with capsule synthesis.

Collectively this suggests that these clones (C3 and C4 isolates) may have evolved from an intestinal strain that has adapted to colonise and survive in the human urinary tract. The redundancy of iron uptake system in clade C4 isolates, similar to that seen in the ST131 clone, confer an adaptive specificity that allows this clone to colonise and survive in different parts of the urinary tract.

## Discussion

Here we report for the first time the detection of carbapenem-resistant UPEC associated with bacteremic UTI cases from Riyadh, KSA. The increased level of antimicrobial resistance identified in the studied isolates is alarming, with the majority of isolates carrying an array of ß-lactamases (against both cephalosporins and carbapenems), and resistance elements to most antibiotics commonly recommended for the treatment of UTIs (e.g. trimethoprim-sulfamethoxazole, ciprofloxacin and ampicillin) [2]. A recent study conducted in Riyadh has demonstrated the high prevalence of ESBL-producing UPEC isolates, particularly CTX-M-15, in hospital- and community- acquired UTIs [44]. Although carbapenem-resistant UPEC were not identified in this study, the authors observed a high level of carbapenem prescription in the community as a consequence of the high prevalence of EBSL-producing bacteria [44]. This is in addition to the higher prevalence of carbapenemase-producing *E*. *coli* and *K*. *pneumonia* isolates recovered from clinical cases from different parts of KSA and the Arabian Peninsula [50–52].

Therefore, the possibility cannot be excluded that these MDR UPEC isolates have been driven by evolutionary pressures associated with the high and indiscriminate use of antimicrobials in the community. The presence of carbapenemase genes in *E*. *coli* in KSA has previously been noted, with the detection of an NDM-positive *E*. *coli* of ST101 in untreated wastewater in Jeddah [53]. While this environmental isolate was able to internalize into mammalian cells and possessed a mosaic of traits representative of different pathotypes, it is unclear if there is ongoing exchange of carbapenemase genes occurring between environmental and clinical isolates of *E*. *coli* or between clinical isolates of *E*. *coli* and other *Enterobacteriaceae*.

The phylogenomic analysis demonstrated that the UPEC isolates from Riyadh are diverse clones that cluster into four distinct UPEC evolutionary clades (ST69 Cplx, ST131 Cplx, ST448 Cplx and ST23 Cplx).The ST131 *E*. *coli* strains have been associated with the global dissemination of carbapenemase-resistant infections [54].

Interestingly, the clade C4 UPEC isolates of ST23 Cplx (ST410) have not previously been identified in KSA, however, ST410 is one of the dominant *E*. *coli* strains in southeast Europe, an area of close proximity to the Middle East. In addition, ST410 was reported in multiple studies in Greece [55], albeit with differences in the genetic makeup of the resistance genes. Recently, *bla*_NDM-5_ and a colistin-resistant *E*. *coli* isolate associated with a complicated UTI case have been reported in the USA [56]. The clade C3 UPEC isolates of ST448 Cplx identified in this study have not been reported within UPEC circulating in KSA nor globally [44]. However, carbapenem-resistant *E coli* ST448 harboring the *bla*_KPC-3_ gene have previously been reported in Europe [57,58]. This might highlight a potential role of Hajj (Muslim pilgrimage to Mecca, KSA that attracts 2 million pilgrims from 188 countries every year [59]) on the introduction and dissemination of MDR-carrying plasmids among different serovars of *E*. *coli*. Recently we have shown that ~40% of *Salmonella* and *E*. *coli*-positive samples associated with Hajj-diarrheal infections carried ESBLs (primarily *bla*_CTX-M-15_) and carbapenemases (*bla*_NDM_) markers [60].

The comparative genomic analysis of the UPEC isolates from Riyadh demonstrates that the phylogenomic clustering is consistent with the isolate ST, pMLST, virulence and AMR signatures. The NDM-1 resistant isolates in clades C1 and C2 (UPEC-C-RIY-1 and UPEC-C-RIY-2, respectively) share an almost complete arsenal of UPEC virulence factors, including different colonization factors, toxins and iron acquisition systems. The NDM-5 resistant isolates belong to clade C4 (UPEC-C-RIY[7–10]) and the clade C3 isolates (UPEC-C-RIY[3–6]) characterized by varied virulence architectures. This is supported by the observation that clade C3 isolates share close evolutionary links with isolates recovered only from urine while clade C4 isolates shared close links with isolates recovered from both urine and blood. Moreover, the clade C4 isolates share multiple iron acquisition systems, including both aerobactin and yersiniabactin, that have been associated with highly virulent ST131 UPEC isolates. Recent studies have shown that aerobactin has higher iron binding capacity than enterobactin [61,62]. Also, yersiniabactin plays an important role in biofilm formation in urine and sequesters host-derived copper, thereby protecting against intracellular killing [63]. The clade C3 isolates, with few major UPEC virulence factors been missed, share multiple ESBL and carbapenemase markers including *bla*_CTX-M-15_, *bla*_TEM-1,_ *bla*_CMY-42_, *bla*_OXA-1_ and *bla*_OXA-181_.

Additionally, comparative genomics demonstrated that both clade C3 and C4 isolates share distinct genomic signatures, including the acquisition of genomic islands associated with intestinal strains, providing some evidence that these clones may have evolved from common intestinal ancestors and then adapted to colonize the urinary tract.

## Conclusions

The analyses demonstrated the existence of fairly diverse clones of carbapenem-resistant UPEC strains in Riyadh, KSA. These results raise major public health concerns, with further research studies, including public and environmental health surveys, using a combination of WGS and phenotyping needed to understand the dynamics of acquisition, transmission and persistence of MDR-UPEC strains in KSA.

## Supporting information

S1 Fig Structural variations identified in UPEC isolates from Riyadh. Artemis Comparison Tool (ACT) views of selected ROD identified in the studied isolates. The structures of ROD2 (A), ROD3 (B), ROD4 (C), ROD8 (D) ROD13 (E) and ROD32 (F) are shown.
(PDF)

S1 Table List of the genomes used in the analysis. (XLSX)

S2 Table SNP patterns identified in chromosomal genes associated with fluoroquinolone resistance. (XLSX)

S3 Table AMR genes identified in UPEC isolates from KSA. (XLSX)

S4 Table Regions of difference identified in UPEC isolates from Riyadh. (XLSX)

S5 Table The distribution of key virulence factors among the studied UPEC isolates. (XLSX)
