# Supplementary figures and images for: Genomic characterization of NDM-1 and 5, and OXA-181 carbapenemases in uropathogenic Escherichia coli isolates from Riyadh, Saudi Arabia
Source: PLoS One. 2018 Aug 15;13(8):e0201613. doi: 10.1371/journal.pone.0201613 (PMC6093660; doi:10.1371/journal.pone.0201613)

A

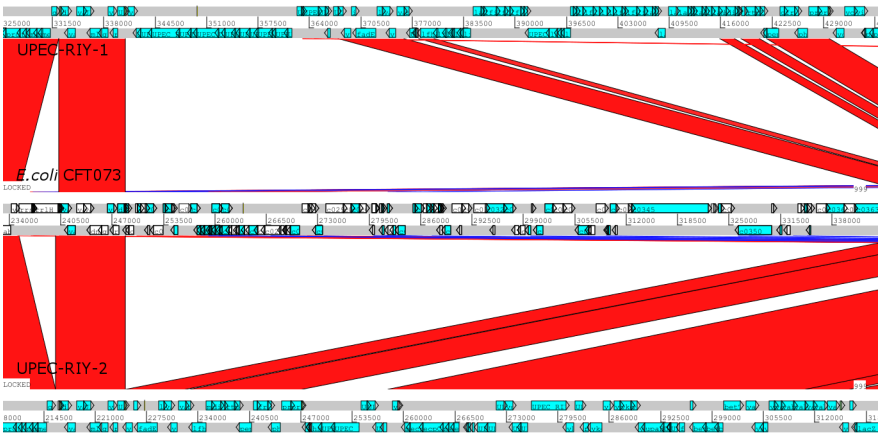

B

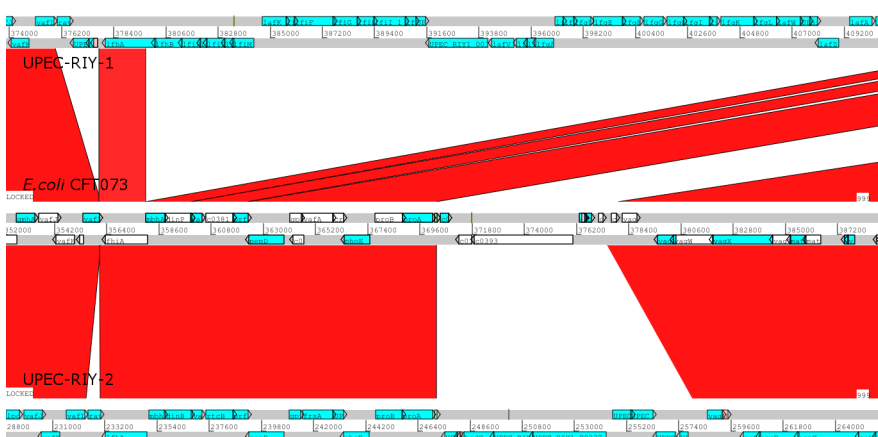

C

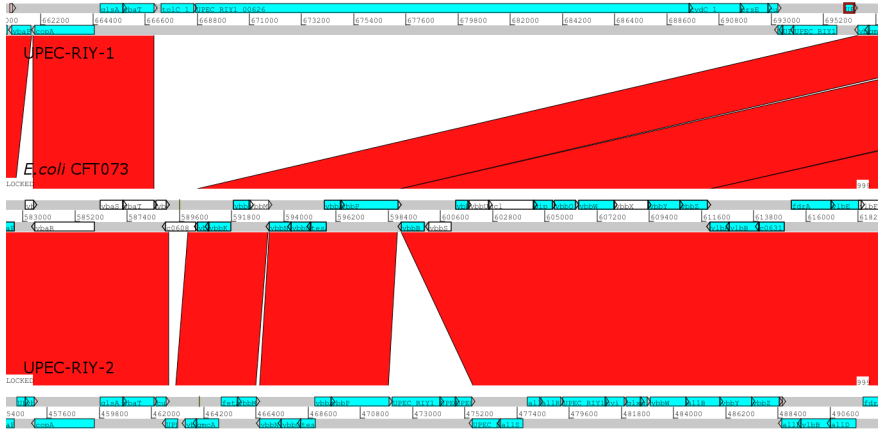

D

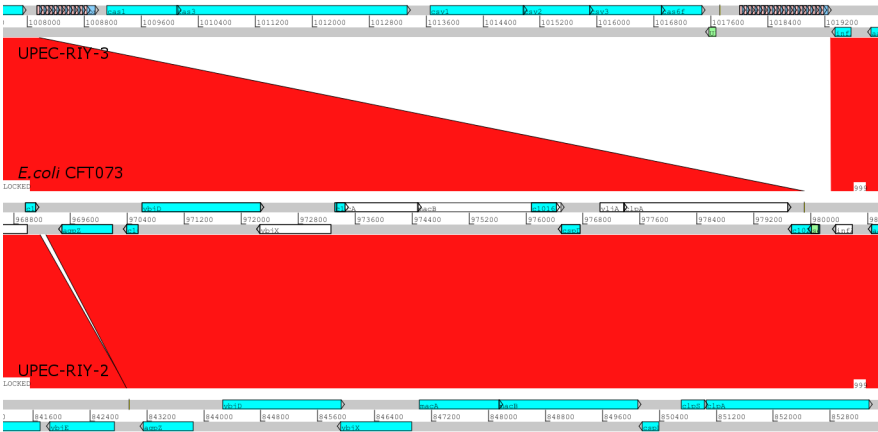

E

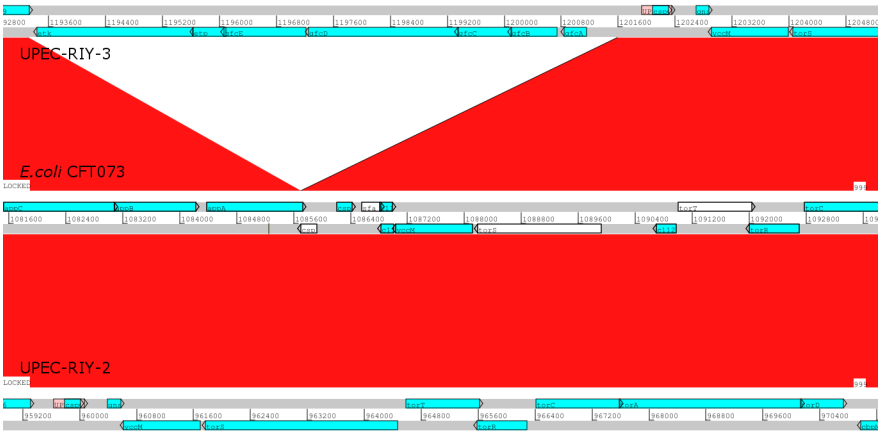

F

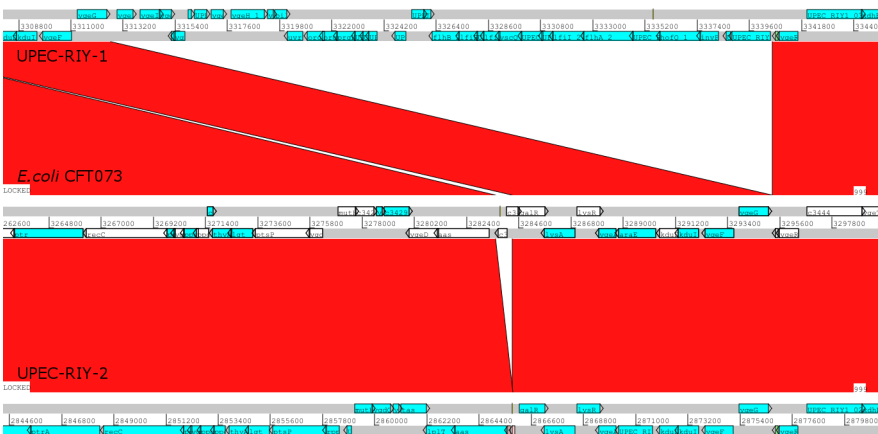

Supplement: S1 Fig — Artemis Comparison Tool (ACT) views of selected ROD identified in the studied isolates. The structures of ROD2 (A), ROD3 (B), ROD4 (C), ROD8 (D) ROD13 (E) and ROD32 (F) are shown. (PDF) [file pone.0201613.s001.pdf]
